# Large optical anisotropy-oriented construction of a carbonate-nitrate chloride compound as a potential ultraviolet birefringent material†

**DOI:** 10.1039/d2sc03771h

**Published:** 2022-11-09

**Authors:** Meng Cheng, Wenqi Jin, Zhihua Yang, Shilie Pan

**Affiliations:** Research Center for Crystal Materials, CAS Key Laboratory of Functional Materials and Devices for Special Environments, Xinjiang Technical Institute of Physics & Chemistry, CAS 40-1 South Beijing Road Urumqi 830011 China slpan@ms.xjb.ac.cn; Center of Materials Science and Optoelectronics Engineering, University of Chinese Academy of Sciences Beijing 100049 China

## Abstract

The design of new birefringent materials is very significant owing to their indispensable role in modulating the polarization of light and is vital in laser technology. Herein, by applying a large optical anisotropy-oriented construction induced by a synergy effect of multiple anionic groups, a promising carbonate-nitrate chloride, Na_3_Rb_6_(CO_3_)_3_(NO_3_)_2_Cl·(H_2_O)_6_, has been designed and synthesized successfully by the solvent evaporation method and single crystals of centimeter size were obtained by the recrystallization method in aqueous solution. It crystallizes in the hexagonal *P*6_3_/*mcm* space group, the RbO_9_Cl polyhedra and the NaO_7_ polyhedra construct a three-dimensional (3D) framework by sharing O or Cl atoms and trigonal plane units (CO_3_ and NO_3_). The transmittance spectrum based on a 1 mm thick single-crystal plate shows that its short UV cut-off edge is about 231 nm. And the refractive index differences (0.14 @ 546 nm) measured by using a polarizing microscope on the (101) crystal plane, proves that Na_3_Rb_6_(CO_3_)_3_(NO_3_)_2_Cl·(H_2_O)_6_ has a large birefringence, which has potential application in the solar blind ultraviolet region. The theoretical calculations reveal that the π-conjugated CO_3_ and NO_3_ groups are the main cause of the birefringence. It demonstrates that combining π-conjugated CO_3_ and NO_3_ groups in one structure is an extremely effective strategy to explore new UV birefringent crystals.

## Abstract 2

This paper reports the first carbonate-nitrate chloride, Na_3_Rb_6_(CO_3_)_3_(NO_3_)_2_Cl·(H_2_O)_6_, with two types of π-conjugated anionic groups, which has been characterized as a new UV birefringent crystal with large birefringence and a short UV cut-off edge.

## Graphical abstract

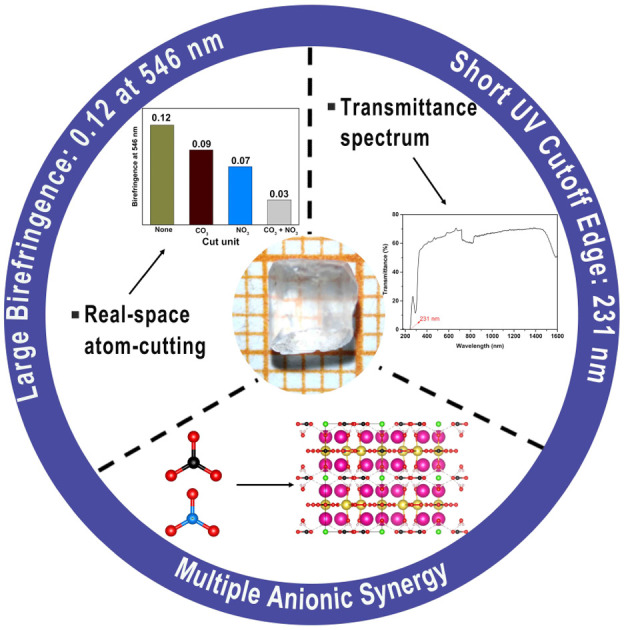

## Introduction

Exploring new birefringent materials is a current academic and technological hotspot due to their applications in polarimetry, optical communication, and scientific instrumentation. Hitherto, several birefringent crystals have been commercialized, such as MgF_2_,^1^ α-BaB_2_O_4_,^2^ CaCO_3_,^3^ and so on, but some drawbacks hinder their practical application in the ultraviolet (UV) including the solar blind ultraviolet region (200–280 nm) or the deep-ultraviolet (DUV, ≤200 nm) region. For example, although a natural calcite crystal (CaCO_3_) with large birefringence (0.17 @ 633 nm) is the most widely used birefringent material for a prism polarizer in the UV to the near–infrared spectral range, its poor optical homogeneity and cleavability limit its application. Thus, designing new UV birefringent crystals is important for scientific and technological research.

Birefringence is the key parameter for birefringent crystals with non-cubic crystal structures, which is generally quantified as the maximum difference between refractive indices exhibited by the material.^4^ Generally, the metal cations and the types of anionic groups play an important role in the magnitude of birefringence. A metal cation centered polyhedron (Pb, Sn, Bi, *etc.*)^5–12^ with a stereochemically active lone pair is beneficial to the generation of high-polarizability anisotropy, which would enhance the optical anisotropy and birefringence of crystals, but they are often impracticable in designing new UV/DUV birefringent crystals since the cut-off edge may be redshifted with these cations. Alkali metal or alkaline-earth metal cations are usually chosen to ensure wide transparency in the UV region, but they generally contribute less to the polarizability anisotropy.^13–22^ Thus, the contribution of anionic groups that are active for birefringence would be crucial in exploring new UV birefringent crystals. Non-π-conjugated anionic groups, *i.e.*, PO_4_ or BO_4_, exhibit a large bandgap, while their relatively small polarizability anisotropy imparts a small birefringence. And it is found that the introduction of fluorine in the tetrahedron can enhance the birefringence or optimize the dispersion of refractive indices, TO_4−*x*_F_*x*_ (T = B, *x* = 1–4; T = P, *x* = 1–2; T = S, and *x* = 1).^23–35^ Planar π-conjugated anionic groups, such as BO_2_, BO_3_, B_3_O_6_, C_3_N_3_O_3_, CO_3_, NO_3_, *etc.*,^35–44^ exhibit strong polarizability anisotropy, which are regarded as the preferred functional units to induce large birefringence. Borates with relatively wide transparency and large birefringence have been studied systematically in recent decades. Among borate birefringent materials, the commercial birefringent material of α-BaB_2_O_4_ shows relatively large birefringence (Δ*n* = 0.12 @ 546 nm)^45^ due to large polarizability anisotropy of B_3_O_6_ anionic groups. Simultaneously, as birefringent crystals, carbonates have emerged in recent years, but nitrates have rarely been reported because they always show water solubility and deliquescence. However, in comparison with BO_3_ and CO_3_ anionic groups, NO_3_ has higher microscopic polarizability anisotropy (Table S5†), and it is easier to obtain large size single crystals, which are useful to further study other properties.

Based on these aspects, our strategy is to introduce multi anionic groups such as CO_3_ and NO_3_ π-conjugated units in one structure to improve the crystal growth of carbonate while maintaining the optical performance of birefringent crystals. Based on the Inorganic Crystal Structure Database (ICSD), there are six examples where CO_3_ and NO_3_ π-conjugated anionic groups coexist in one structure. Except for the [Pb_6_O_4_](OH)(NO_3_)(CO_3_)^46^ compound, other compounds are transition metal (Pt or Co) amine complexes ((Pt(NH_3_)_2_)_4_(CO_3_)_2_)(NO_3_)_4_·(H_2_O)_3_,^47^ (Co(NH_3_)_4_(CO_3_)(NO_3_))_2_·H_2_O,^48^ (Co(CO_3_)(NH_3_)_4_)(NO_3_),^49^ (Co(NH_3_)_4_CO_3_)(NO_3_)·(H_2_O)_0.5_ ^50^ and (Co(NH_3_)_5_(CO_3_))(NO_3_)·H_2_O.^51^ In this work, we combine two types of π-conjugated anionic groups and a halogen anion in one compound. And Na_3_Rb_6_(CO_3_)_3_(NO_3_)_2_Cl·(H_2_O)_6_, the first carbonate-nitrate chloride has been successfully synthesized by the solvent evaporation method. And the crystal growth was carried out by the recrystallization method, and the single crystals of centimeter size were obtained. Herein, the details of synthesis and crystal growth, the IR spectrum, thermal behavior, the UV-vis-NIR diffuse-reflectance spectrum, the transmittance spectrum and the refractive index difference of Na_3_Rb_6_(CO_3_)_3_(NO_3_)_2_Cl·(H_2_O)_6_ are reported. Simultaneously, we analyzed the influence mechanism of multiple anionic units by first-principles calculations. The real space atom-cutting method and electron density difference map were employed to analyze the origin of the large birefringence. The experimental and theoretical results show that Na_3_Rb_6_(CO_3_)_3_(NO_3_)_2_Cl·(H_2_O)_6_ is a potential UV birefringent crystal with a large birefringence and short UV cut-off edge.

## Experimental

### Synthesis of the single crystal

Na_3_Rb_6_(CO_3_)_3_(NO_3_)_2_Cl·(H_2_O)_6_ was synthesized by the solvent evaporation method through reacting NaHCO_3_ (10 mmol, 0.8401 g), RbNO_3_ (10 mmol, 1.4747 g), RbCl (10 mmol, 1.2092 g) and deionized water. The mixture was stirred at room temperature and the solution turned colorless and transparent, and then the solution was evaporated at room temperature. Finally, transparent and block crystals grew out in 3 days. As shown in Fig. S1,† single crystals of centimeter size were obtained by the recrystallization method in aqueous solution in 14 days.

### Characterization

A coreless single crystal with suitable size of the title compound was selected for single crystal X-ray diffraction. All diffraction data were collected on a Bruker D8 Venture with Mo Kα (*λ* = 0.71073 Å) at 298(2) K. The intensity, reduction, and cell refinement investigations were carried out on a Bruker SAINT.^52^

All the structures were solved by a direct method and refined through the full-matrix least-squares fitting on *F*^2^ with SHELX^53^ and OLEX2^54^ software. PLATON^55^ was used to confirm the higher symmetry. Crystallographic data and further details for structural analyses are listed in Table 1, and the selected bond distances are summarized in Table S2.† Powder X-ray diffraction (PXRD) data were collected by putting the powder sample onto flat sample holders utilizing a Bruker D2 Phaser X-ray diffractometer equipped with Cu-Kα radiation (*λ* = 1.54056 Å) and the diffraction patterns were taken in the range from 10 to 70° (2 theta). The powder XRD patterns of pure polycrystalline samples exhibit good consistency with the calculated XRD ones. The calculated XRD patterns were produced by the Mercury v3.8 program and their single-crystal structure data. Polycrystalline samples used for thermogravimetric (TG) analysis and differential scanning calorimetry (DSC) were ground from bulk crystals directly. Infrared spectroscopy was carried out on a Shimadzu IR Affinity-1 Fourier transform IR spectrometer with a resolution of 2 cm^−1^ in the 500 to 4000 cm^−1^ range. The polycrystalline samples were mixed thoroughly with dried KBr with a mass ratio of about 1 (polycrystalline sample) : 100 (KBr). A Shimadzu Solid Spec-3700 DUV spectrophotometer was used to collect the UV-vis-NIR diffuse-reflectance data for the title compounds. The spectrophotometer worked ranging from 175 to 2600 nm at room temperature. The transmittance spectrum was measured using a transparent crystal plate with 1 mm thickness in the 175–1600 nm wavelength range. Absorption data (*K*/*S*) were worked out from the following Kubelka–Munk^56^ function:1
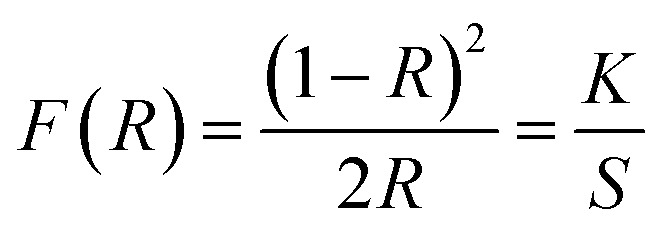
in which *K* is the absorption, *R* is the reflectance, and *S* is the scattering. Extrapolating the linear part of the sloping upward curve to zero in the (*K*/*S*)-*versus-E* plot yields the appearance of absorption. The refractive index difference of the title compound was characterized by using a polarization microscope equipped (ZEISS Axio Scope. 5 pol) with a Berek compensator. The average wavelength of the light source was 546 nm. The formula for calculating the birefringence is listed below,

2 *R* = |*N*_g_ − *N*_p_| × *d* = Δ*n* × *d*

Here, *R* represents the optical path difference; *N*_g_, *N*_p_ and Δ*n* mean the refractive index of fast light, slow light, and the difference value of the refractive index, respectively; *d* denotes the thickness of the crystal.

**Table 1:** Crystal data and structure refinement for Na_3_Rb_6_(CO_3_)_3_(NO_3_)_2_Cl·(H_2_O)_6_

Empirical formula	Na_3_Rb_6_(CO_3_)_3_(NO_3_)_2_Cl·(H_2_O)_6_
Formula weight	1029.39
Temperature	298 (2) K
Wavelength	0.71073 Å
Crystal system	Hexagonal
Space group	*P*6_3_/*mcm*
Unit cell dimensions	*a* = 9.5732(3) Å
	*c* = 15.8202(11) Å
Volume	1255.62(10) Å^3^
*Z*	2
Density (calculated)	2.723 Mg m^−3^
Absorption coefficient	11.849 mm^−1^
*F*(000)	968
Crystal size	0.17 × 0.16 × 0.11 mm^3^
Theta range for data collection	2.457 to 27.541°
Index ranges	−10 ≤ *h* ≤ 12, −12 ≤ *k* ≤ 12, −20 ≤ *l* ≤ 20
Reflections collected	10 235
Independent reflections	557 [*R*(int) = 0.0775]
Completeness to theta = 27.541°	100.0%
Refinement method	Full-matrix least-squares on *F*^2^
Data/restraints/parameters	557/0/44
Goodness-of-fit on *F*^2^	1.074
Final *R* indices [*I* > 2sigma(*I*)]^a^	*R*_1_ = 0.0238, w*R*_2_ = 0.0457
*R* indices (all data)^a^	*R*_*1*_ = 0.0393, w*R*_2_ = 0.0511
Largest diff. peak and hole	0.436 and −0.484 e Å^−3^

a *R*_1_ = ∑‖*F*_o_| − |*F*_c_‖/∑|*F*_o_| and w*R*_2_ = [∑w(*F*_o_^2^ − *F*_c_^2^)^2^/∑w*F*_o_^4^]^1/2^ for *F*_o_^2^ > 2*σ*(*F*_o_^2^).

### Theoretical calculations

The electronic structure was calculated using density functional theory (DFT) performed using the plane wave pseudopotential implemented in the CASTEP package.^57^ Using the norm-conserving pseudopotential (NCP),^58^ the explicitly treated valence electrons for each atom were calculated as follows: Na, 3s^1^; Rb, 5s^1^; C, 2s^2^2p^2^; N, 2s^2^2p^3^; H, 1s^1^, O, 2s^2^2p^4^, and Cl, 3s^2^3p^5^. The exchange–correlation functional was the Perdew–Burke–Ernzerhof (PBE) functional within the generalized gradient approximation (GGA).^59,60^ The TS method was used for the DFT-D correction.^61^ The plane-wave energy cutoff was set at 750.0 eV. The separation of the *k*-point was set as 0.03 Å in the Brillouin zone. The number of empty bands were set as 3 times of valence bands for the calculation of the optical properties. Because the GGA method always underestimates the bandgap, the scissors operators were utilized to shift the conduction bands so that they agree with the experimental bandgap values, and then the refractive indices were obtained by the real part of the dielectric function on the base of the Kramers–Kronig transform. The HOMO–LUMO energy gap and polarizability anisotropy (*δ*) of anionic groups were calculated using DFT implemented by the Gaussian09 package.^62^ The B3LYP (Becke, three-parameter, Lee–Yang–Parr) exchange–correlation functional with the Lee–Yang–Parr correlation functional at the 6-31G basis set in Gaussian was employed.

## Results and discussion

### Crystal structure

The crystallographic analysis reveals that Na_3_Rb_6_(CO_3_)_3_(NO_3_)_2_Cl·(H_2_O)_6_ (CCDC 2181425) crystallizes in the space group *P*6_3_/*mcm* (no. 193) of the hexagonal crystal system. There is one independent Na (6g), one independent Rb (12k), two independent C (4d and 2a), one independent N (4c), four independent O (12i, 12j, 12k, and 6g), one independent Cl (2b) and one independent H (24l) wyckoff sites (Table S1†) in the asymmetric unit. In the structure of the title compound, there are two different π-conjugated anionic groups of CO_3_ and NO_3_. In the structure, the Na–O bond lengths vary from 2.268(4) to 2.715(4) Å. The two C atoms are three-coordinated in a π-conjugated planar triangle with the C–O lengths in the range of 1.277(4)–1.280(3) Å. The N atoms are three-coordinated in a π-conjugated planar triangle with the N–O bond lengths of 1.250(3) Å. The Rb–O bond lengths vary from 3.030(2) to 3.401(3) Å and the Rb–Cl bond length is 3.2282(4) Å. The O–C–O and O–N–O bond angles are of the 3-fold symmetrical 120° angle (Table S2†). The water molecule plays the role of a hydrogen bond donor, and the O(2) atom of carbonate ions plays the role of a hydrogen bond acceptor. The distance between O(2) and O(4) atoms is 2.702(5) Å and the angle of O(3)–H⋯O(2) is 176(3) °. Each carbonate ion connects with six water molecules by hydrogen bond interaction. Furthermore, bond valence sum (BVS) calculations give values of 1.22, 1.10, 4.91, 4.08, 4.04, 2.09, 1.60, 0.45, 2.08, and 1.26 for Na(1), Rb(1), N(1), C(1), C(2), O(1), O(2), O(3), O(4), and Cl(1), which are consistent with oxidation states of 1, 1, 5, 4, 2, and 1 for Na, Rb, N, C, non-hydroxyl O, and Cl, respectively. The calculated BVS for O(2) is 1.60, since the hydrogen bond interaction can be found among O(2), O(3) and H atoms, *i.e.*, O(3)–H⋯O(2). The calculated BVS for O(3) is 0.45, suggesting that this is for H_2_O groups, which was also confirmed by the IR data.

**Fig. 1:**
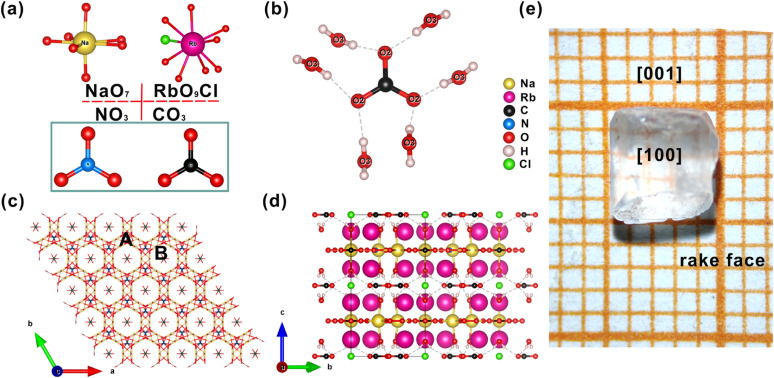
(a) The NaO_7_, RbO_9_Cl, CO_3_ and NO_3_ groups; (b) hydrogen bonds between water molecules and carbonate ions; (c) the wireframe of the title compounds in the *ab* plane; (d) the structure of the title compounds in the *bc* plane; (e) photograph of an as-grown Na_3_Rb_6_(CO_3_)_3_(NO_3_)_2_Cl·(H_2_O)_6_ crystal.

For the alkali metal atoms, as indicated in Fig. 1a, the Na atoms are coordinated by seven O atoms to form a NaO_7_ pentagonal bipyramid and the Rb atoms are coordinated by nine O atoms and one Cl atom to form an RO_9_Cl polyhedron. The Rb-centered polyhedron and the NaO_7_ polyhedron construct a three-dimensional (3D) framework by sharing O or Cl atoms with each other. As shown in Fig. 1c, there are two types (9-membered ring channel (type A) and 6-membered ring channel (type B)) of channels in the 3D framework. In the framework, C(2)O_3_ and N(1)O_3_ trigonal plane units reside in 9-membered channels and Cl atoms and C(1)O_3_ trigonal plane units reside in 6-membered channels. The NaO_7_ pentagonal bipyramid and C(1)O_3_ and N(1) O_3_ trigonal plane units form a 2D layer and stack along the [001] direction. Simultaneously, owing to the directivity and saturation of hydrogen bonds, C(2)O_3_ trigonal plane units and water molecules are connected by hydrogen bonding forces to further stabilize the structure. For clarity, the Rb–O/Cl bonds are deleted, Fig. 1d shows the total structure of title compounds in the *bc* plane. As shown in Fig. 1e, the photograph of an as-grown Na_3_Rb_6_(CO_3_)_3_(NO_3_)_2_Cl·(H_2_O)_6_ crystal is consistent with the theoretical crystal morphology (Fig. S6†), and the crystal orientation is determined by using an X-ray crystal orientation instrument.

### Optical properties and birefringence

In order to further confirm the coordination environments of anionic groups in the structure, IR spectroscopic measurements were carried out (see Fig. S2b†). The characteristic vibration frequency of the O–H bond is 3500–3730 cm^−1^. Affected by hydrogen bonds (O–H⋯Y), H is pulled over by Y, and the characteristic vibration frequency will be reduced to 3100–3500 cm^−1^. Therefore, the broad IR absorption bands at ∼3160 cm^−1^ confirm the presence of water molecules and hydrogen bonds (O–H⋯O) in the structure. The broad and strong absorption bands in the range of 1451–1367 cm^−1^ and 883–660 cm^−1^ can be assigned to the characteristic absorption of the NO_3_ or CO_3_ groups. These results are in accordance with those of previously reported studies. The UV-vis-NIR diffuse reflectance spectrum of Na_3_Rb_6_(CO_3_)_3_(NO_3_)_2_Cl·(H_2_O)_6_ is shown in Fig. S2a.† Its UV cut-off absorption edge is at about 220 nm, which demonstrates that the title compound is a UV optical material. To further confirm the UV cut-off edge, the crystal was grown for the UV-vis-NIR transmittance spectrum measurement, and the result based on a 1 mm thick preliminary polishing single-crystal plate shows that it has a UV cut-off edge of 231 nm.

**Fig. 2:**
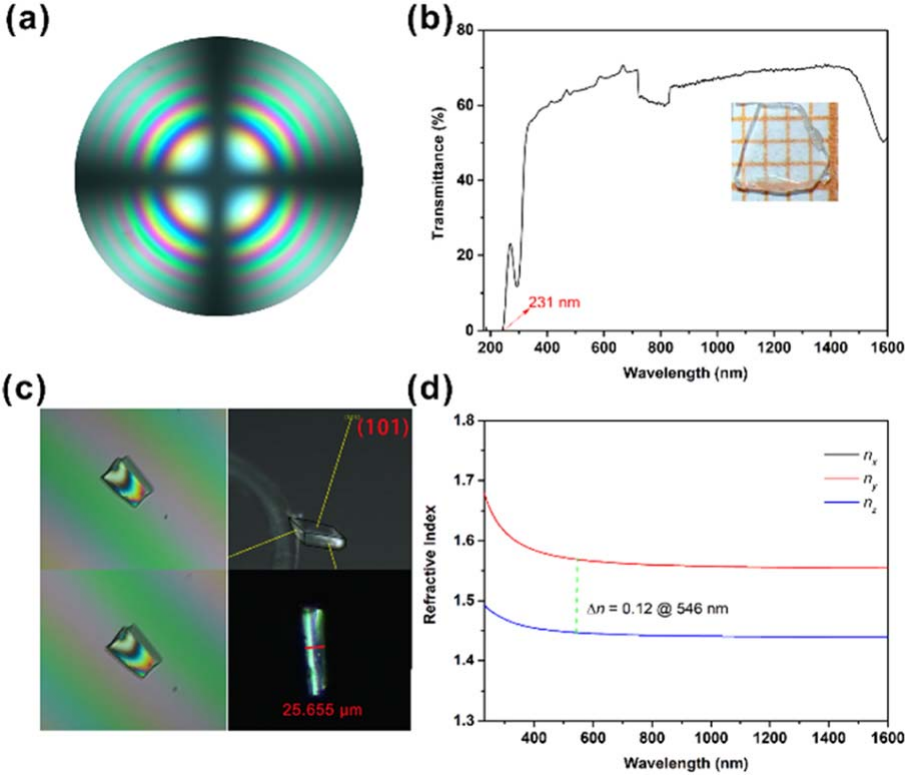
(a) Interference pattern of polarized light; (b) transmittance spectrum of Na_3_Rb_6_(CO_3_)_3_(NO_3_)_2_Cl·(H_2_O)_6_; (c) positive (up) and negative (down) compensatory rotation, and the thickness of the (101) plate-crystal; (d) calculated refractive index dispersion curves.

It is well-known that the planar triangular groups of CO_3_ and NO_3_ have large polarizability anisotropy. Therefore, the crystal may show a large birefringence when the CO_3_ and NO_3_ groups are stacked parallelly to form a perfect layered structure. The calculated linear optical results of Na_3_Rb_6_(CO_3_)_3_(NO_3_)_2_Cl·(H_2_O)_6_ show that *n*_*x*_ = *n*_*y*_ > *n*_*z*_, that is, *n*_o_ > *n*_e_, indicating that the title compound is a negative uniaxial optical crystal. The interference pattern of polarized light indicates that Na_3_Rb_6_(CO_3_)_3_(NO_3_)_2_Cl·(H_2_O)_6_ is a uniaxial crystal (Fig. 2a). According to the calculated refractive index dispersion curves, its birefringence is 0.12 at 546 nm (Fig. 2d), which is comparable to that of α-BBO. The refractive index difference was measured under the polarizing microscope method, using (101) crystal planes. The crystal thickness of the title compound is 25.655 μm. And the optical path differences at 546 nm are 12.75 and 12.95 μm. According to the formula, the refractive index differences on the (101) crystal planes can be calculated to be 0.14 @ 546 nm, which indicates that Na_3_Rb_6_(CO_3_)_3_(NO_3_)_2_Cl·(H_2_O)_6_ may have a birefringence larger than 0.14 @ 546 nm.

### Electronic structure and mechanism of birefringence

**Fig. 3:**
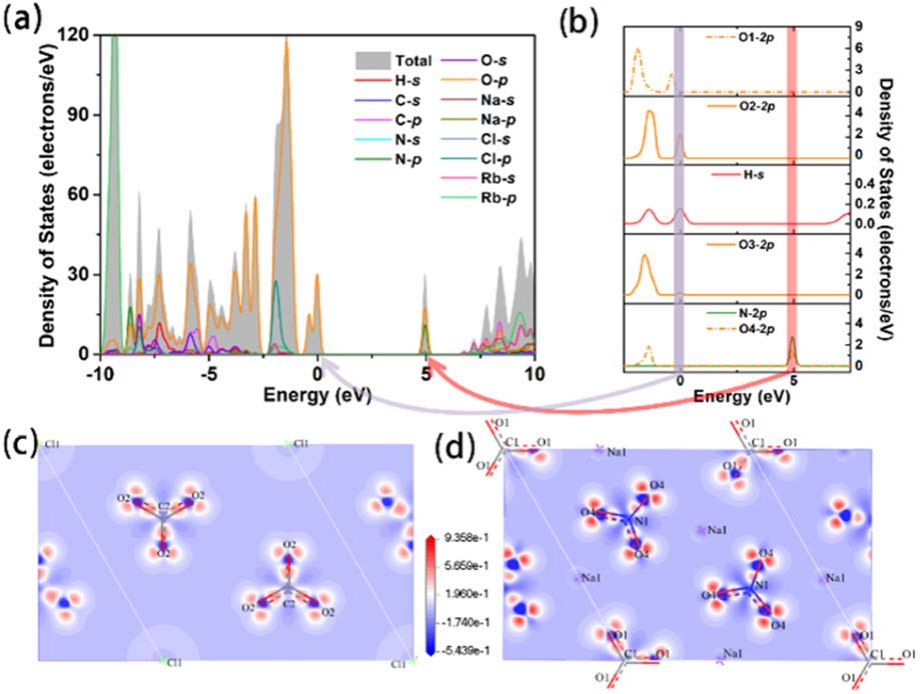
(a) Total density of states; (b) partial density of states of O and H atoms; (c and d) electron density difference of CO_3_ and NO_3_ groups.

In order to get more insight into the interaction between the microstructure and optical properties for Na_3_Rb_6_(CO_3_)_3_(NO_3_)_2_Cl·(H_2_O)_6_, systematic theoretical calculations on the basis of density functional theory (DFT) were used. The partial densities of states (PDOS) reflect the distribution of atomic orbitals. The valence bands (VBs) are mainly composed of H-s, C-p, N-p, O-p, Cl-p and Rb-p, and the conductive bands (CBs) mainly originate from H-s, C-p, N-p, O-p, Na-sp and Rb-sp. The top of the VB, quite close to the Fermi level, is occupied by O-p and H-s; with further analysis of each crystallographically independent O atom together with a H atom, a clearer hydrogen bond interaction can be found among O(2), O(3) and H atoms, *i.e.*, O(3)-H⋯O(2) (Fig. 3b), which is similar to the hydrogen bond in paracetamol.^63^ At the bottom of the CB, several separate electronic states of N-2p, O-2p (specifically, O(4)-2p, Fig. 3b) and Na-p appear, among which the interaction between N-2p and O(4)-2p means a NO_3_ group. These separate electronic structures at the bottom of the CB were also reported in other nitrates.^64,65^ Therefore, the bandgap is mainly determined by the hydrogen bond, Na-p, N-p and O-p.

In order to identify the origin of the large birefringence, the real space atom-cutting (RSAC)^66^ method and electron density difference map^67^ are employed. From the calculated electron density difference map of CO_3_ and NO_3_ (Fig. 3c and d), we can see that there are obvious covalent characteristics between the C–O or N–O bonds. As shown in Table S6,† the main contribution comes from the CO_3_ and NO_3_ anionic units. To evaluate the contribution of hydrogen bonds to birefringence, H_2_O was cut off from the structure, and the calculated birefringence is also unchanged (Table S6†). In this case, the calculated birefringence is 0.12 @ 546 nm. Thus, we speculate that hydrogen bonds do not contribute significantly to the birefringence of the title compound. Based on the calculated results which are listed in Table S6,† the planar π-conjugated CO_3_ and NO_3_ anionic units play a major role in the large birefringence.

## Conclusions

In conclusion, the first carbonate-nitrate chloride, Na_3_Rb_6_(CO_3_)_3_(NO_3_)_2_Cl·(H_2_O)_6_, with two types of π-conjugated anionic groups has been characterized as a new UV birefringent crystal with large birefringence and a short UV cut-off edge. For Na_3_Rb_6_(CO_3_)_3_(NO_3_)_2_Cl·(H_2_O)_6_, the RbO_9_Cl and NaO_7_ polyhedra construct a three-dimensional framework by sharing O or Cl atoms, and the planar π-conjugated groups (CO_3_ and NO_3_) reside in a 9-membered ring channel and 6-membered ring channel, respectively in the 3D framework. And the hydrogen bond interaction between CO_3_ and H_2_O will further stabilize the structure. We have obtained single crystals of centimeter size by a simple and environmentally friendly aqueous solution method. Simultaneously, we analyze the influence mechanism of multiple anionic units by first-principles calculations. The result shows that the planar π-conjugated CO_3_ and NO_3_ anionic units play a major role in the large birefringence. The experimental refractive index difference is 0.14 @ 546 nm on the (101) crystal plane, which is comparable to that of the α-BBO (0.12 @ 546 nm) crystal. The synthesis and investigation of other new alkaline-metal carbonate-nitrate compounds with excellent properties are still in progress.

## Data availability

All of the related experimental and computational data are provided in the ESI.†

## Author contributions

M. C. and S. L. P. designed the research study; M. C. synthesized the compound and performed the experiments. W. Q. J. and Z. H. Y. performed the optical theoretical calculations. All authors wrote and revised the manuscript. All the authors contributed to the final manuscript preparation.

## Conflicts of interest

The authors declare no competing financial interests.

## Supplementary Material

SC-013-D2SC03771H-s001

SC-013-D2SC03771H-s002

## References

[cit1] Sedlmeir F., Zeltner R., Leuchs G., Schwefel H. G. L. (2014). Opt. Express.

[cit2] Zhou G. Q., Xu J., Chen X. D., Zhong H. Y., Wang S. T., Xu K., Deng P. Z., Gan F. X. (1998). J. Cryst. Growth.

[cit3] Ghosh G. (1999). Opt. Commun..

[cit4] Niu S., Joe G., Zhao H., Zhou Y., Orvis T., Huyan H., Salman J., Mahalingam K., Urwin B., Wu J., Liu Y., Tiwald T. E., Cronin S. B., Howe B. M., Mecklenburg M., Haiges R., Singh D. J., Wang H., Kats M. A., Ravichandran J. (2018). Nat. Photonics.

[cit5] Tang R. L., Hu C. L., Mao F. F., Feng J. H., Mao J. G. (2019). Chem. Sci..

[cit6] Guo J., Tudi A., Han S., Yang Z., Pan S. (2019). Angew. Chem., Int. Ed..

[cit7] Jing Q., Yang Z., Pan S., Xue D. (2015). Phys. Chem. Chem. Phys..

[cit8] Guo J., Tudi A., Han S., Yang Z., Pan S. (2021). Angew. Chem., Int. Ed..

[cit9] Han S., Mutailipu M., Tudi A., Yang Z., Pan S. (2020). Chem. Mater..

[cit10] Dong X., Jing Q., Shi Y., Yang Z., Pan S., Poeppelmeier K. R., Young J., Rondinelli J. M. (2015). J. Am. Chem. Soc..

[cit11] Kim K., Lee M. H., Ok K. M. (2021). Chem. Mater..

[cit12] Zou G., Ok K. M. (2020). Chem. Sci..

[cit13] Chen X. L., Zhang B. B., Zhang F. F., Wang Y., Zhang M., Yang Z., Poeppelmeier K. R., Pan S. L. (2018). J. Am. Chem. Soc..

[cit14] Bai Z., Liu L., Wang D., Hu C. L., Lin Z. (2021). Chem. Sci..

[cit15] Mutailipu M., Zhang M., Yang Z. H., Pan S. L. (2019). Acc. Chem. Res..

[cit16] Mutailipu M., Pan S. (2020). Angew. Chem., Int. Ed..

[cit17] Zhang W., Zhang Z., Jin W., Zhang R., Cheng M., Yang Z., Pan S. (2021). Sci. China. Chem..

[cit18] Li Z., Lin Z., Wu Y., Fu P., Wang Z., Chen C. (2004). Chem. Mater..

[cit19] Han G., Lei B., Yang Z., Wang Y., Pan S. (2018). Angew. Chem., Int. Ed..

[cit20] Li S., Liu X., Wu H., Song Z., Yu H., Lin Z., Hu Z., Wang J., Wu Y. (2021). Chem. Sci..

[cit21] Li Z., Jin W., Zhang F., Chen Z., Yang Z., Pan S. (2022). Angew. Chem., Int. Ed..

[cit22] Zhang B., Shi G., Yang Z., Zhang F., Pan S. (2017). Angew. Chem., Int. Ed..

[cit23] Cheng H., Li F., Yang Z., Pan S. (2022). Angew. Chem., Int. Ed..

[cit24] Mutailipu M., Zhang M., Wu H., Yang Z., Shen Y., Sun J., Pan S. (2018). Nat. Commun..

[cit25] Zhang B., Han G., Wang Y., Chen X., Yang Z., Pan S. (2018). Chem. Mater..

[cit26] Lu J., Yue J. N., Xiong L., Zhang W. K., Chen L., Wu L. M. (2019). J. Am. Chem. Soc..

[cit27] Jin W., Zhang W., Tudi A., Wang L., Zhou X., Yang Z., Pan S. (2021). Adv. Sci..

[cit28] Wang Y., Zhang B., Yang Z., Pan S. (2018). Angew. Chem., Int. Ed..

[cit29] Wu H., Yu H., Pan S., Huang Z., Yang Z., Su X., Poeppelmeier K. R. (2013). Angew. Chem., Int. Ed..

[cit30] Wang X., Wang Y., Zhang B., Zhang F., Yang Z., Pan S. (2017). Angew. Chem., Int. Ed..

[cit31] Shi G., Wang Y., Zhang F., Zhang B., Yang Z., Hou X., Pan S., Poeppelmeier K. R. (2017). J. Am. Chem. Soc..

[cit32] Mutailipu M., Zhang M., Zhang B., Wang L., Yang Z., Zhou X., Pan S. (2018). Angew. Chem., Int. Ed..

[cit33] Zhang Z. Z., Wang Y., Zhang B. B., Yang Z. H., Pan S. L. (2018). Angew. Chem., Int. Ed..

[cit34] Yang Z., Lei B., Zhang W., Pan S. (2019). Chem. Mater..

[cit35] Zhao J., Ma Y., Li R. (2015). Appl. Opt..

[cit36] Cao L., Tian H., Lin D., Lin C., Xu F., Han Y., Yan T., Chen J., Li B., Ye N., Luo M. (2022). Chem. Sci..

[cit37] Huang C., Mutailipu M., Zhang F., Griffith K. J., Hu C., Yang Z., Griffin J. M., Poeppelmeier K. R., Pan S. (2021). Nat. Commun..

[cit38] Kang K., Liang F., Meng X., Tang J., Zeng T., Yin W., Xia M., Lin Z., Kang B. (2019). Inorg. Chem..

[cit39] Chen Z., Zeng H., Han S., Yang Z., Pan S. (2021). New J. Chem..

[cit40] Liu X., Kang L., Gong P., Lin Z. (2021). Angew. Chem., Int. Ed..

[cit41] Zhang X., Guo L., Zhang B., Yu J., Wang Y., Wu K., Wang H. j., Lee M. H. (2021). Chem. Commun..

[cit42] Cheng B., Li Z., Chu Y., Tudi A., Mutailipu M., Zhang F., Yang Z., Pan S. (2022). Natl. Sci. Rev..

[cit43] Guo S., Jiang X., Liu L., Xia M., Fang Z., Wang X., Lin Z., Chen C. (2016). Chem. Mater..

[cit44] Liu H., Wang Y., Zhang B., Yang Z., Pan S. (2020). Chem. Sci..

[cit45] Solntsev V. P., Tsvetkov E. G., Gets V. A., Antsygin V. D. (2002). J. Cryst. Growth.

[cit46] Li Y. P., Krivovichev S. V., Burns P. C. (2000). J. Solid State Chem..

[cit47] Mahtani H. K., Chang S. C., Ruble J. R., Black I. N. L., Stein P. B. (1993). Inorg. Chem..

[cit48] Bernal I., Cetrullo J. (1990). Struct. Chem..

[cit49] Le Bail A. (2013). Acta Crystallogr., Sect. E: Struct. Rep. Online.

[cit50] Christensen A. N., Hazell R. G. (1999). Acta Chem. Scand..

[cit51] Zhu J. H., Wu H. X., Le Bail A. (1999). Solid State Sci..

[cit52] SAINT, version 7.60A, Bruker Analytical X-ray Instruments, Inc., Madison, WI, 2008

[cit53] Sheldrick G. M. , SHELXTL, version 6.14, Bruker Analytical X-ray Instruments, Inc. Madison, WI, 2008

[cit54] Dolomanov O. V., Bourhis L. J., Gildea R. J., Howard J. A. K., Puschmann H. (2009). J. Appl. Crystallogr..

[cit55] Spek A. L. (2003). J. Appl. Crystallogr..

[cit56] Kubelka P., Munk F. (1931). Zeit. Tekn. Physik..

[cit57] Sheldrick G. M. (2015). Acta Crystallogr., Sect. C: Cryst. Struct. Commun..

[cit58] Rappe A. M., Rabe K. M., Kaxiras E., Joannopoulos J. D. (1990). Phys. Rev. B: Condens. Matter Mater. Phys..

[cit59] Lin J. S., Qteish A., Payne M. C., Heine V. (1993). Phys. Rev. B: Condens. Matter Mater. Phys..

[cit60] Perdew J. P., Burke K., Ernzerhof M. (1997). Phys. Rev. Lett..

[cit61] Tkatchenko A., Scheffler M. (2009). Phys. Rev. Lett..

[cit62] Frisch M. J. , Trucks G. W., Schlegel H. B., Scuseria G. E., Robb M. A., Cheeseman J. R., Scalmani G., Barone V., Mennucci B., Petersson G. A., Nakatsuji H., Caricato M., Li X., Hratchian H. P., Izmaylov A. F., Bloino J., Zheng G., Sonnenberg J. L., Hada M., Ehara M., Toyota K., Fukuda R., Hasegawa J., Ishida M., Nakajima T., Honda Y., Kitao O., Nakai H., Vreven T., Montgomery Jr. J. A., Peralta J. E., Ogliaro F., Bearpark M., Heyd J. J., Brothers E., Kudin K. N., Staroverov V. N., Kobayashi R., Normand J., Raghavachari K., Rendell A., Burant J. C., Iyengar S. S., Tomasi J., Cossi M., Rega N., Millam J. M., Klene M., Knox J. E., Cross J. B., Bakken V., Adamo C., Jaramillo J., Gomperts R., Stratmann R. E., Yazyev O., Austin A. J., Cammi R., Pomelli C., Ochterski J. W., Martin R. L., Morokuma K., Zakrzewski V. G., Voth G. A., Salvador P., Dannenberg J. J., Dapprich S., Daniels A. D., Farkas O., Foresman J. B., Ortiz J. V., Cioslowski J. and Fox D. J., Gaussian 09, Revision A.02, Gaussian, Inc., Wallingford CT, 2009

[cit63] An G. W., Zhang H., Cheng X. L., Zhuo Q. L., Lv Y. C. O. (2008). Struct. Chem..

[cit64] Korzhneva K. E., Bekenev V. L., Khyzhun O. Y., Goloshumova A. A., Tarasova A. Y., Molokeev M. S., Isaenko L. I., Kurus A. F. (2021). J. Solid State Chem..

[cit65] Korabel’nikov D. V., Zhuravlev Y. N. (2015). J. Phys. Chem. Solids.

[cit66] Lin J., Lee M. H., Liu Z. P., Chen C. T., Pickard C. J. (1999). Phys. Rev. B: Condens. Matter Mater. Phys..

[cit67] Lei B., Pan S., Yang Z., Cao C., Singh D. J. (2020). Phys. Rev. Lett..

