# Supplementary material for: Large optical anisotropy-oriented construction of a carbonate-nitrate chloride compound as a potential ultraviolet birefringent material
Source: Chem Sci. 2022 Nov 9;13(45):13482–8. doi: 10.1039/d2sc03771h (PMC9685371; doi:10.1039/d2sc03771h)
Supplement: SC-013-D2SC03771H-s001 [file SC-013-D2SC03771H-s001.pdf]

Electronic Supplementary Material (ESI) for Chemical Science.
This journal is © The Royal Society of Chemistry 2022

Supporting information

Large Optical Anisotropy-Oriented Construction of Carbonate-Nitrate Chloride as Potential Ultraviolet Birefringent Materials

Meng Cheng, [ab] Wenqi Jin, [ab] Zhihua Yang, [ab] Shilie Pan*[ab]
[a]Research Center for Crystal Materials; CAS Key Laboratory of Functional Materials and Devices for Special Environments; Xinjiang Technical Institute of Physics and Chemistry, CAS, 40-1 South Beijing Road, Urumqi 830011, China.
[b]Center of Materials Science and Optoelectronics Engineering, University of Chinese Academy of Sciences, Beijing 100049, China.
*Corresponding author: slpan@ms.xjb.ac.cn

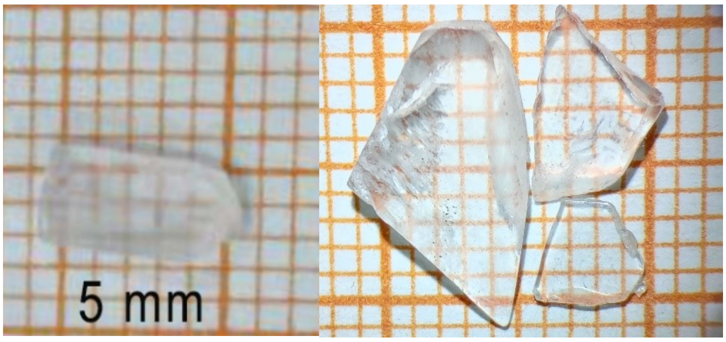

Figure S1. Photograph of crystals grown via mild solvent evaporation method.

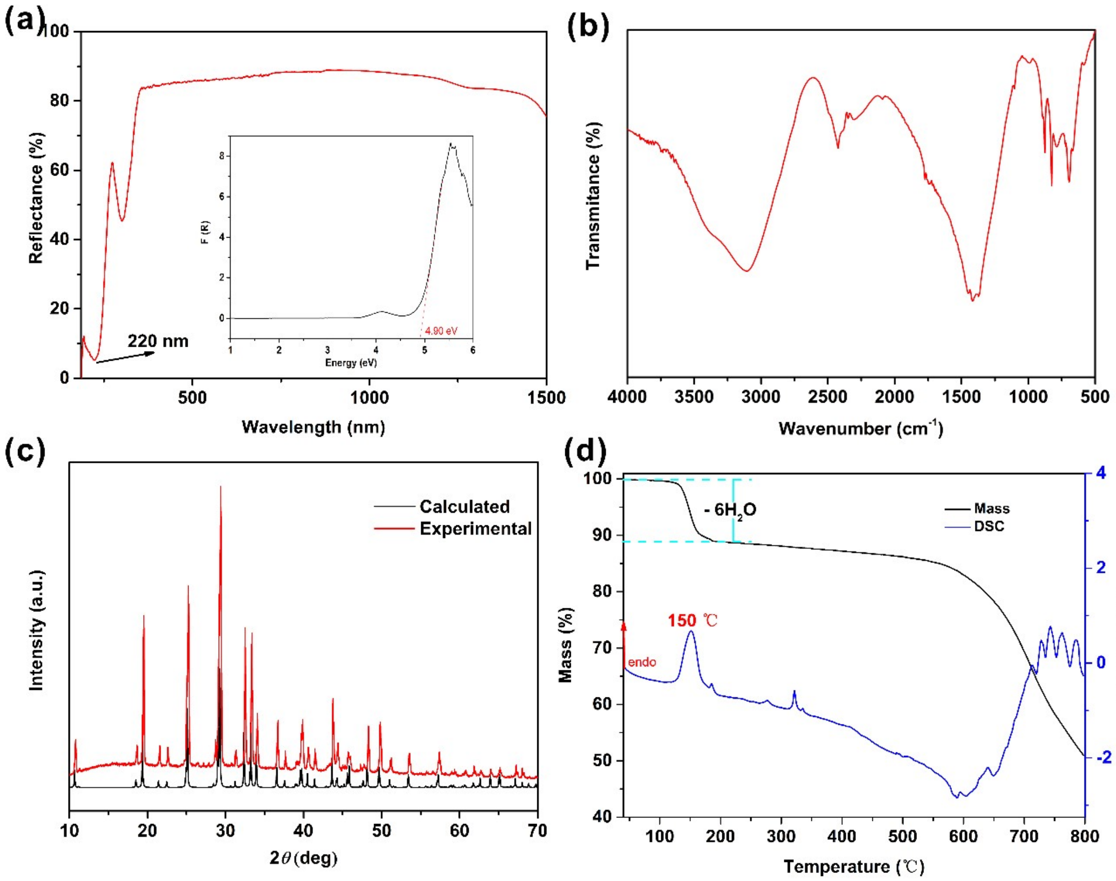

Figure S2. (a) UV−vis-NIR diffuse reflectance spectra for $Na_3Rb_6(CO_3)_3(NO_3)_2Cl\cdot(H_2O)_6$; (b) IR spectrum for $Na_3Rb_6(CO_3)_3(NO_3)_2Cl\cdot(H_2O)_6$; (c) XRD patterns; (d) TG and DSC curves of $Na_3Rb_6(CO_3)_3(NO_3)_2Cl\cdot(H_2O)_6$ in the temperature region from 40 to 800 °C.

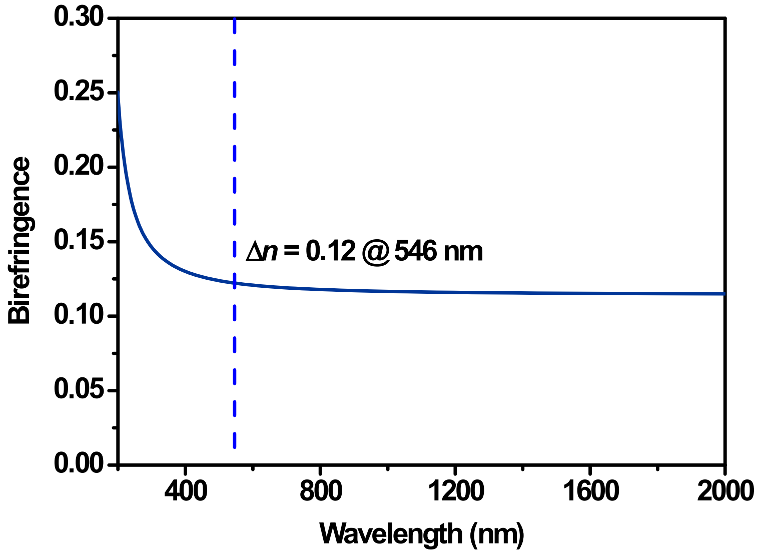

Figure S3. Calculated birefringence curve.

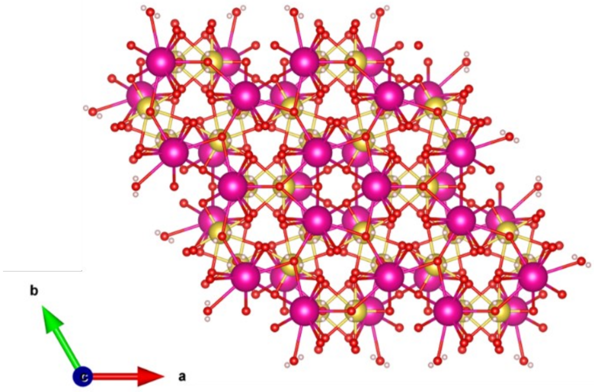

Figure S4. The structure of the title compounds in the *ab* plane excluding C, N and Cl atoms.

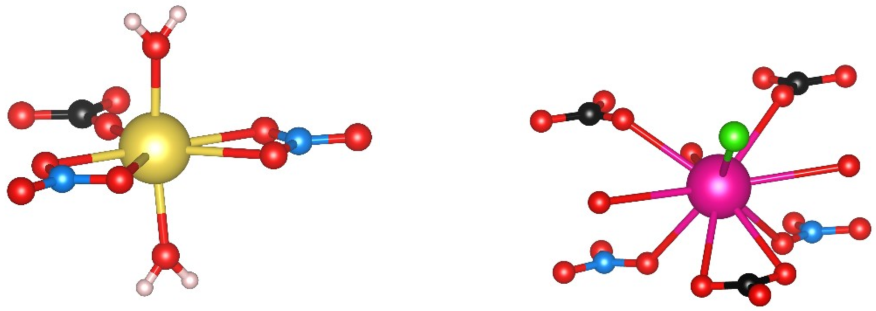

Figure S5. Alignments of planar triangular anion groups by the $NaO_5(H_2O)_2$ and RbO_9Cl polyhedra.

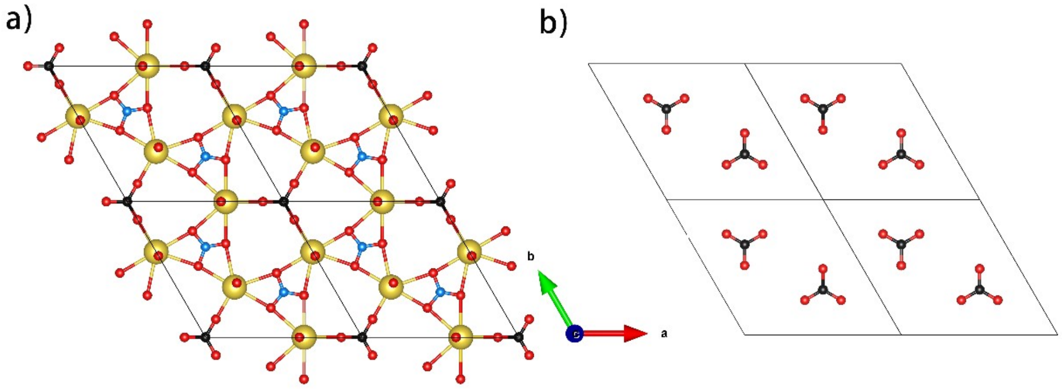

Figure S6. a) Two-dimensional (2D) layer of NaO_7 pentagonal bipyramid, C(1)O_3 and NO_3 trigonal plane units; b) The C(2)O_3 trigonal plane units in the *ab* plane.

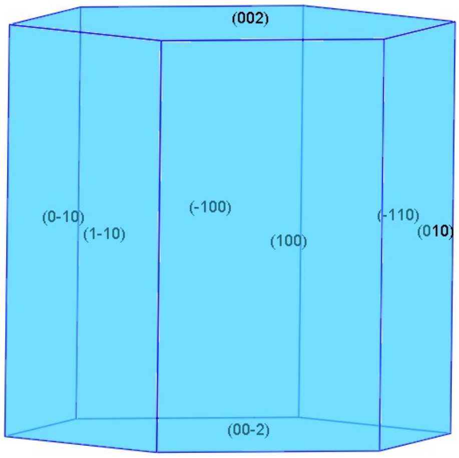

Figure S7. Theoretical crystal morphology of $Na_3Rb_6(CO_3)_3(NO_3)_2Cl\cdot(H_2O)_6$.

Table S1. Atomic coordinates (×10[4]), equivalent isotropic displacement parameters (Å^2×10^3) and bond valence sum (BVS) calculation for $Na_3Rb_6(CO_3)_3(NO_3)_2Cl\cdot(H_2O)_6$. U(eq) is defined as one third of the trace of the orthogonalized U_{ij} tensor.

	**Wyckoff sites**	***x***	***y***	***z***	***U(eq)***	**BVS**
Na(1)	6g	3703(2)	0	2500	24(1)	1.22
Rb(1)	12k	2737(1)	2737(1)	1192(1)	27(1)	1.10
C(1)	2a	0	0	2500	16(2)	4.08
C(2)	4d	6667	3333	0	24(2)	4.04
N(1)	4c	6667	3333	2500	22(1)	4.91
O(1)	6g	0	1334(4)	2500	28(1)	2.09
O(2)	12j	5123(4)	2562(2)	0	33(1)	1.60
O(3)	12k	5997(4)	5997(4)	1053(2)	36(1)	0.45
O(4)	6g	5256(4)	3088(4)	2500	34(1)	2.08
Cl(1)	2b	0	0	0	29(1)	1.26

Table S2. Selected bond distances (Å) and angles (deg) for $Na_3Rb_6(CO_3)_3(NO_3)_2Cl\cdot(H_2O)_6$.

**Bond**	**Distances (Å)**
Na(1)-O(4)	2.560(3)
Na(1)-O(4)#4	2.715(4)
Na(1)-O(4)#11	2.560(3)
Na(1)-O(4)#12	2.715(4)
Na(1)-O(3)#4	2.307(3)
Na(1)-O(3)#13	2.307(3)
Na(1)-O(1)#6	2.268(4)
Rb(1)-Cl(1)	3.2282(4)
Rb(1)-O(4)	3.066(2)
Rb(1)-O(4)#2	3.066(2)
Rb(1)-O(2)	3.030(2)
Rb(1)-O(2)#3	3.030(2)
Rb(1)-O(3)#4	3.400(3)
Rb(1)-O(3)	3.128(4)
Rb(1)-O(3)#5	3.400(3)
Rb(1)-O(1)	3.0718(4)
Rb(1)-O(1)#6	3.0718(4)
C(1)-O(1)#1	1.277(4)
C(1)-O(1)	1.277(4)
C(1)-O(1)#6	1.277(4)
C(2)-O(2)#4	1.280(3)
C(2)-O(2)#17	1.280(3)
N(1)-O(4)#15	1.250(3)
N(1)-O(4)#4	1.250(3)

**Bond**	**Angles (deg)**	**Bond**	**Angles (deg)**
O(4)#4-Na(1)-O(4)#12	82.91(13)	O(2)#3-Rb(1)-Cl(1)	85.41(4)
O(4)#11-Na(1)-O(4)#12	48.36(13)	O(2)-Rb(1)-O(4)#2	132.40(7)
O(4)-Na(1)-O(4)#12	131.27(8)	O(2)#3-Rb(1)-O(4)#2	81.55(5)
O(4)-Na(1)-O(4)#4	48.36(13)	O(2)-Rb(1)-O(4)	81.55(5)
O(4)#11-Na(1)-O(4)#4	131.27(8)	O(2)#3-Rb(1)-O(4)	132.40(7)
O(4)-Na(1)-O(4)#11	179.63(17)	O(2)#3-Rb(1)-O(2)	88.984(13)
O(3)#13-Na(1)-O(4)#12	84.62(8)	O(2)#3-Rb(1)-O(3)	66.93(5)
O(3)#13-Na(1)-O(4)#4	84.62(8)	O(2)-Rb(1)-O(3)#4	49.30(5)
O(3)#13-Na(1)-O(4)#11	89.977(11)	O(2)-Rb(1)-O(3)	66.93(5)
O(3)#4-Na(1)-O(4)#11	89.977(11)	O(2)#3-Rb(1)-O(3)#4	135.74(6)

O(3)#4-Na(1)-O(4)#4	84.62(8)	O(2)#3-Rb(1)-O(3)#5	49.30(5)
O(3)#4-Na(1)-O(4)#12	84.62(8)	O(2)-Rb(1)-O(3)#5	135.74(6)
O(3)#4-Na(1)-O(4)	89.977(11)	O(2)#3-Rb(1)-O(1)#6	153.72(7)
O(3)#13-Na(1)-O(4)	89.977(11)	O(2)-Rb(1)-O(1)#6	113.09(6)
O(3)#13-Na(1)-O(3)#4	165.6(2)	O(2)-Rb(1)-O(1)	153.72(7)
O(1)#6-Na(1)-O(4)#12	138.55(7)	O(2)#3-Rb(1)-O(1)	113.09(6)
O(1)#6-Na(1)-O(4)#4	138.55(7)	O(3)-Rb(1)-Cl(1)	140.24(6)
O(1)#6-Na(1)-O(4)#11	90.19(8)	O(3)-Rb(1)-O(3)#4	101.65(4)
O(1)#6-Na(1)-O(4)	90.19(8)	O(3)#5-Rb(1)-O(3)#4	154.95(9)
O(1)#6-Na(1)-O(3)#4	97.18(11)	O(3)-Rb(1)-O(3)#5	101.65(4)
O(1)#6-Na(1)-O(3)#13	97.18(11)	O(1)#6-Rb(1)-Cl(1)	82.51(3)
Cl(1)-Rb(1)-O(3)#5	78.12(5)	O(1)-Rb(1)-Cl(1)	82.51(3)
Cl(1)-Rb(1)-O(3)#4	78.12(5)	O(1)-Rb(1)-O(3)#5	63.81(8)
O(4)#2-Rb(1)-Cl(1)	139.29(6)	O(1)-Rb(1)-O(3)	133.72(7)
O(4)-Rb(1)-Cl(1)	139.29(6)	O(1)#6-Rb(1)-O(3)#5	105.15(8)
O(4)-Rb(1)-O(4)#2	71.74(10)	O(1)#6-Rb(1)-O(3)#4	63.81(8)
O(4)#2-Rb(1)-O(3)#4	135.11(7)	O(1)-Rb(1)-O(3)#4	105.15(8)
O(4)-Rb(1)-O(3)#5	135.11(7)	O(1)#6-Rb(1)-O(3)	133.72(7)
O(4)#2-Rb(1)-O(3)	66.45(7)	O(1)#6-Rb(1)-O(1)	42.18(13)
O(4)#2-Rb(1)-O(3)#5	64.18(7)	O(1)-C(1)-O(1)#1	120
O(4)-Rb(1)-O(3)	66.45(7)	O(1)-C(1)-O(1)#6	120.000(1)
O(4)-Rb(1)-O(3)#4	64.18(7)	O(1)#1-C(1)-O(1)#6	120
O(4)-Rb(1)-O(1)#6	67.88(8)	O(2)#4-C(2)-O(2)	120.000(1)
O(4)#2-Rb(1)-O(1)#6	92.59(4)	O(2)#4-C(2)-O(2)#17	120
O(4)-Rb(1)-O(1)	92.59(4)	O(4)#4-N(1)-O(4)#15	120.000(1)
O(4)#2-Rb(1)-O(1)	67.88(8)	O(4)#4-N(1)-O(4)	120
O(2)-Rb(1)-Cl(1)	85.41(4)	O(4)#15-N(1)-O(4)	120

Symmetry transformations used to generate equivalent atoms:

# 1 x-y,x,-z	#2 y,x,z	# 3-y,x-y,z
# 4 -x+y,-x+1,z	#5 -y+1,x-y,z	# 6-x,-y,-z
# 7 y,-x+y,-z	#8 -x+y,-x,z	# 9-y+1,x-y,-z+1/2
# 10 x-y,-y,-z+1/2	#11 -x+1,-x+y,z	# 12y,x-1,z
# 13 x,y,-z+1/2	#14 -x+y,-x,-z+1/2	# 15-x+y+1,-x+1,-z+1/2
# 16 -y+1,x-y+1,z	# 17-x+y+1,-x+1,z	# 18-y,x-y,-z+1/2

Table S3. Anisotropic displacement parameters for $Na_3Rb_6(CO_3)_3(NO_3)_2Cl\cdot(H_2O)_6$.

	U_{11}	U_{22}	U_{33}	U_{23}	U_{13}	U_{12}
Na(1)	21(1)	24(1)	26(1)	0	0	12(1)
Rb(1)	26(1)	26(1)	28(1)	0(1)	0(1)	11(1)
C(1)	21(3)	21(3)	6(4)	0	0	10(2)
C(2)	26(2)	26(2)	19(4)	0	0	13(1)
N(1)	21(2)	21(2)	25(3)	0	0	10(1)
Cl(1)	27(1)	27(1)	31(1)	0	0	14(1)
O(1)	33(2)	19(2)	35(3)	0	0	16(1)
O(2)	24(2)	30(1)	44(2)	4(1)	0	12(1)
O(3)	46(2)	46(2)	26(2)	-3(1)	-3(1)	31(2)
O(4)	19(2)	39(2)	48(2)	0	0	16(1)

Table S4. Hydrogen bonds for $Na_3Rb_6(CO_3)_3(NO_3)_2Cl\cdot(H_2O)_6$.

D-H···A	d(D-A)/Å	D-H···A/°
O(3)-H(3)···O(2)#1	2.702(5)	176(3)

Symmetry transformations used to generate equivalent atoms:
1 1-x+y,1-x,z

Table S5. HOMO-LUMO energy gap and polarizability anisotropy of BO_3, NO_3, and CO_3.

	**Energy gap (eV)**	**δ**
**BO_3**	7.84	9.45
**NO_3**	5.39	17.67
**CO_3**	7.36	12.80

Table S6. Real-space atom-cutting analysis of calculated birefringence in $Na_3Rb_6(CO_3)_3(NO_3)_2Cl{\cdot}(H_2O)_6$. The cutting radii of Na, Rb, C, N, O (CO_3, NO_3, H_2O), Cl, and H were set as 0.76, 0.94, 0.28, 0.63, (1.01, 0.63, 0.72) and 0.27 Å, respectively.

**Cut unit**	**Birefringence （@ 546 nm）**
None	0.12
H_2O	0.12
Na, Rb, Cl, and H_2O	0.14
CO_3	0.09
NO_3	0.07
$CO_3 + NO_3$	0.03
